# Correlation between COVID-19-related health anxiety and coping styles among frontline nurses

**DOI:** 10.1186/s12912-023-01344-3

**Published:** 2023-07-10

**Authors:** Maryam Saeedi, Zahra Abedini, Maryam Latif, Masoumeh Piruzhashemi

**Affiliations:** 1 Saveh University of Medical Sciences, Saveh, Iran; 2 Department of Nursing, School of Nursing and Midwifery, Qom University of Medical Sciences, Qom, Iran; 3 Shahid Chamran Hospital, Saveh, Iran; 4 Department of Hefdah Shahrivar and Shahid Modares Hospital, Saveh University of Medical Sciences, Saveh, Iran

**Keywords:** Anxiety, Adaptation, psychological, Nurses, COVID-19

## Abstract

**Background:**

The long-term epidemic of COVID-19 and its recurrence has exposed frontline nurses to mental disorders such as stress and health anxiety. High levels of health anxiety associated with COVID-19 may lead to maladaptive behaviors. There is no agreement on which coping styles are most effective against stress. Therefore, more evidence is required to find better adaptive behaviors. The present study was conducted to investigate the correlation between the level of health anxiety and the type of coping strategies used by frontline nurses fighting COVID-19.

**Methods:**

This is a cross-sectional study that was performed on a convenience sample of 386 nurses who are working in the COVID department from October to December 2020, coinciding with the outbreak of the third COVID-19 peak in Iran. Data were collected through a demographic questionnaire, the short version of the health anxiety questionnaire, and coping inventory for stressful situations. Data were analyzed using independent T-test, U-Mann-Whitney, and Kruskal-Wallis statistical tests with SPSS version 23 software.

**Results:**

The mean score of nurses’ health anxiety was 17.61 ± 9.26 which was more than the cut-off point for health anxiety and 59.1% of nurses had COVID-19-related health anxiety. The mean score of problem coping style (26.85 ± 5.19), was higher than emotion (18.48 ± 5.63) and avoidance (19.64 ± 5.88) coping styles and nurses mostly used problem-coping strategies to deal with anxiety caused by COVID-19. There was a positive significant correlation between the scores of health anxiety and emotion coping style (r = 0.54; P < 0.001).

**Conclusion:**

Based on the findings of this study, COVID-19-related health anxiety was high in front-line nurses and those with high health anxiety were more likely to use emotion-based coping strategies, which are ineffective. Therefore, considering strategies to reduce nurses’ health anxiety and holding training programs on effective coping methods in epidemic conditions are recommended.

## Background

The outbreak of the COVID-19 epidemic showed our unpreparedness due to its sudden and rapid spread, which confused many governments around the world [1]. Because of the prevalence of COVID-19 worldwide and its mortality rate, healthcare workers are more likely than others to be exposed to psychological disorders due to being on the front line of the disease [2]. Among health workers, nurses have higher anxiety and stress [3, 4].

Front-line nursing staff suffer from psychological distress due to close interaction with patients [5, 6]. One of the psychological issues that have plagued frontline nurses during the COVID-19 outbreak is health anxiety [7–9]. In one study, the prevalence of health anxiety in nurses caring for COVID-19 patients was reported more than 90% [9]. The high prevalence of health anxiety in nurses requires special attention.

Health anxiety is characterized by a high level of fear and anxiety about having a serious illness [10]. Health anxiety is a continuous concept that on the one hand is a constant health concern, and on the other hand is hypochondriasis, which is characterized by extreme fears about health and sometimes physical symptoms [11]. Almost everyone experiences some degree of health anxiety. Awareness of potential health threats can be protective and help identify early signs of illness and promote healthy behaviors, but if health anxiety is excessive, it can be harmful [12].

Health anxiety varies from person to person. In Iranian society, 43.8% had mild to moderate anxiety and 19.1% had serious anxiety related to COVID-19 [13]. Health anxiety is more prevalent among healthcare workers than in the general population [14]. The prevalence of COVID-19-related health anxiety in healthcare workers has been reported 30.14 to 72.3% [15–20].

The high prevalence of health anxiety among health personnel, especially at the beginning of the Covid-19 epidemic, was caused by the unfamiliar nature of the disease, the lack of medical equipment and personal safety, the high workload, overcrowding of patients, and the fear of transmitting the disease to their families [20–22].

High levels of health anxiety can lead to depression, headaches, insomnia, and suicidal thoughts [7], and it can seriously affect professional performance [23]. Therefore, it is important to deal with the stress caused by this disorder. New perspectives on stress emphasize the role of individual psychological resources in dealing with stressors; because the more appropriate methods are used to deal with stress, the less damage will be done to the person. Applying appropriate coping strategies can prevent the occurrence and continuation of stressful states and restlessness in the person [24].

Coping is defined as the thoughts and actions that individuals use to manage stressful situations [25]. There are three strategies for coping with stress, including problem-oriented, emotion-oriented, and avoidance-oriented coping styles.

Problem-oriented coping style is the direct cognitive actions of an individual that are performed to change and correct threatening environmental conditions and occurs in situations that are controllable or changeable for the individual. Examples of problem-oriented strategies are using past experiences, realism, gathering more information to solve the problem, consulting, and paying attention to the positive points of the problem. Emotion-oriented coping style is used in situations where the person feels that the situation is out of control. This style includes preoccupation, daydreaming, anger, crying, loneliness, depression, and other conscious activities. The avoidance strategy is based on escape from the problem and manifests itself in the form of turning to the community and engaging in a new activity [26, 27].

There is no agreement on which coping styles are most effective against stress, and to what extent a coping strategy helps solve problems and alleviate emotional distress. However, the previous research indicated that emotion-focused coping strategies in long term may be less adaptive than problem-focused strategies, although the effectiveness of these coping strategies styles depends on the stressful situation [28–30].

Studies emphasize the role of coping strategies on COVID-19-related anxiety and mental health. The results of a review study showed that the use of positive coping strategies such as seeking social support, positive thinking, and problem-solving was associated with lower levels of stress and psychological distress, anxiety, and depression caused by COVID-19. While the use of avoidance strategies has been associated with higher psychological stress [31–33].

In most studies, there is no agreement on the type of coping strategies for people who had high health anxiety related to COVID-19. Religious coping [34], engaging with social media [35], wishful thinking [36], using emotional social support, and behavioral disengagement [37], were reported as the dominant coping strategies in people with high COVID-19-related health anxiety.

Some studies have been conducted in Iran to investigate health anxiety or coping strategies separately or together in healthcare workers [38–40], but no study has investigated the relationship between health anxiety and coping strategies in frontline nurses.

High levels of health anxiety associated with COVID-19 may lead to maladaptive behaviors to reduce anxiety. Therefore, more evidence is required to find better adaptive behaviors [41]. Effective coping strategies are important for stressful situations such as the COVID-19 epidemic because they can prevent stress-related psychiatric disorders [42].

It can be assumed that frontline nurses have health anxiety due to direct contact with COVID-19 patients. Nurses with health anxiety may use a specific coping strategy more often.

Considering the importance of using appropriate coping strategies in reducing COVID-19-related health anxiety, The present study was conducted to investigate the correlation between the level of health anxiety and the type of coping strategies used by frontline nurses fighting COVID-19. This study was designed to answer the following questions: (1) How prevalent is health anxiety among front-line nurses who take care of COVID-19 patients? (2) What is the level of health anxiety in these nurses? (3) What coping strategy is most used by front-line nurses in the condition of COVID-19? (4) What is the dominant coping strategy in nurses who have health anxiety? This study hypothesizes that there is a significant correlation between the scores of various coping styles (including problem-oriented, emotion-oriented, and avoidance-oriented) and the health anxiety score of frontline nurses.

## Methods

### Design

This is a cross-sectional study that was performed on nurses who were working in the COVID department and directly caring for patients with COVID-19 from October to December 2020, coinciding with the outbreak of the third COVID-19 peak in Iran.

### Participants

The study samples included nurses working in selected hospitals of Saveh and Tehran (two cities in Iran) by non-probability convenience sampling method. Sampling and data collection were done by two nurse researchers. The researchers visited the COVID department of the selected hospitals in person and in compliance with the health protocols related to COVID. After stating the objectives and methods of the research, the present nurses were asked to complete the written informed consent and the research questionnaires. Samples were included in the study if they desired to participate in the study and completed the informed consent form to participate in the study, were directly involved in patient care in the COVID department during the epidemic, and had more than one year of nursing experience. The samples were excluded from the study if they did not complete the study questionnaires.

The sample size was computed using the statistical program G * Power 3.1.9.2. Based on the small effect size F2 of 0.05, error probability (alpha) of 5%, and power of 95%, a minimum sample size of 210 was calculated. Due to sample availability, 386 nurses were included in this research, of which 325 completed the questionnaires. The response rate was 84.2%.

### Measures

#### Data were collected through 3 questionnaires as follows

1- Demographic information questionnaire: The questions included age, gender, marital status, having children, having underlying disease, level of education, type of hospital, and work experience.

2- The short version of the Health Anxiety Questionnaire: this questionnaire was developed by Salkovskis and Warwick and consists of 18 items. Each item has four options and each option has a score between 0 and 3. The total score of the questionnaire is from 0 to 54, and higher scores indicate a higher level of anxiety [43]. Also, the cut-off point for health anxiety is 15, and a score of 15 and above means having health anxiety [16]. The questionnaire has a two-factor structure including the illness likelihood (14 questions) and the negative consequences of getting the disease (4 questions). Cronbach’s alpha coefficient of the questionnaire was reported between 0.7 and 0.92 by the designer [43]. This questionnaire has been validated in Persian. In these studies, Cronbach’s alpha coefficient of the questionnaire was calculated from 0.75 to 0.95, which indicates the acceptable reliability of the Persian version of the questionnaire [44–46]. The convergent validity of the questionnaire was 0.74 with Beck Depression Inventory [44].

3- The short form of the Coping Inventory for Stressful Situations (CISS): this questionnaire has 21 items and evaluates the coping strategies of individuals in stressful situations from three dimensions including problem-oriented, emotion-oriented, and avoidance-oriented coping styles [26]. This questionnaire is self-report and the subjects should determine on a five-point Likert scale how much they use of each of the presented strategies and the dominant style is determined according to the scores. In each of the coping styles, Cronbach’s alpha coefficients for the subscales of problem-oriented coping are 0.87 − 0.78, emotion-oriented coping is 0.87 − 0.78 and avoidance coping is 0.8 − 0.7 [27]. The CISS has been validated in Persian. In these studies, Cronbach’s alpha coefficient of the questionnaire was calculated from 0.7 to 0.97, which is acceptable [47–49].

### Data analysis

Statistical analyses were performed using SPSS software version 23. Descriptive statistical methods such as determining the mean, standard deviation, and frequency were used to describe the demographic variables and the scores of health anxiety and coping styles. To check the normality of data, Kolmogorov–Smirnov test was used. Statistical tests including the independent T-test, U-Mann-Whitney, and Kruskal-Wallis tests were used to compare the mean scores of health anxiety and coping styles based on demographic variables. The Spearman correlation coefficient was used to evaluate the correlation between nurses’ health anxiety scores and coping styles.

### Ethical review

The study protocol was approved by the Ethics Committee of Saveh University of Medical Sciences (Code: IR.SAVEHUMS.REC.1399.008) and complied with the requirements of the Helsinki Declaration. Before data collection, the objectives and methods of the research were explained to the participants. Participants were assured that their information and responses would be kept confidential and anonymous. They were assured that participation in the study was voluntary. Written informed consent was obtained from the participants before completing the questionnaire.

## Results

The demographic information of the samples is presented in Table 1. The majority of the samples were female (79.4%), in the age range of 31–40 years (41.7%), married (75%), had children (62.2%) and bachelor’s degree (76.4%), worked in public hospitals (71.6%), had a work experience of 1–10 years (47.5%), and no underlying disease (86.8%).

**Table 1:** Demographic characteristics of the participants

Variables	Category	N (%)
Gender	Female Male	258(79.4) 67(20.6)
Age category	20–30 31–40 41–50 51–60	90(28) 134(41.7) 81(25.2) 16(5)
Marital status	Single Married	81(25) 243(75)
Having children	Yes No	202(62.2) 123(37.7)
Level of education	Undergraduate Bachelor MA and above	24(7.4) 249(76.4) 53(16.3)
Having disease	Yes No	43(13.2) 282(85.6)
Work experience (years)	1–10 11–20 21–30 > 30	153(47.5) 124(38.5) 43(13.4) 2(0.6)
Type of hospital	Public Private Semiprivate	232(71.6) 24(7.4) 68(21)

The results of the Kolmograph-Smirnov test to check the normality of the data showed that the variables of health anxiety, problem-oriented, and emotion-oriented coping styles did not have a normal distribution, and the avoidance-oriented coping style had a normal distribution.

The mean score of nurses’ health anxiety was 17.61 ± 9.26 which was more than the cut-off point for health anxiety and 59.1% of nurses had COVID-19-related health anxiety. The mean score of problem coping style (26.85 ± 5.19), was higher than emotion (18.48 ± 5.63) and avoidance (19.64 ± 5.88) coping styles and nurses mostly used problem coping strategies to deal with anxiety caused by COVID-19 (Table 2).

**Table 2:** The comparison of nurses’ coping styles based on having health anxiety

Having health anxiety	Problem style Mean ± SD	Emotion style Mean ± SD	Avoidance style Mean ± SD
Yes	26.69 ± 5.36	20.67 ± 5.06	19.29 ± 5.71
No	27.01 ± 5	15.41 ± 4.82	20.06 ± 6.15
Total	26.85 ± 5.19	18.48 ± 5.63	19.64 ± 5.88
P-value	0.65**	0.000**	0.27*

* Independent T-test

** U- Mann-Whitney test

The comparison of nurses’ coping strategies and health anxiety showed that the mean score of emotion–oriented coping style was significantly (P < 0.001) higher in nurses who had health anxiety than in nurses without health anxiety (Table 2). There was a positive significant correlation between the scores of health anxiety and emotion coping style (r = 0.54; P < 0.001). There was no significant correlation between health anxiety and avoidance and problem-oriented coping styles (Table 3).

**Table 3:** Correlation between nurses’ health anxiety scores and coping styles

Coping style	r *	P
Problem	0.01	0.84
Emotion	0.54	0.000
Avoidance	-0.04	0.54

* Spearman correlation coefficient

The comparison of health anxiety scores and coping strategies based on demographic characteristics showed that health anxiety was significantly associated with age and work experience, problem-oriented strategies were significantly associated with age, having children, level of education, and type of hospital, and emotion-based strategies was significantly associated with age (Table 4).

**Table 4:** The comparison of health anxiety and Coping styles score based on the demographic characteristics of the participants

Variables		Health anxiety Mean ± SD	Problem Mean ± SD	Emotion Mean ± SD	Avoidance Mean ± SD
Age category	20–30 31–40 41–50 51–60 P-value**	18.17 ± 9.02 18.81 ± 9.62 15.41 ± 8.61 13.8 ± 6.03 0.02	25.33 ± 5.05 26.88 ± 5.48 28.47 ± 4.42 27.8 ± 5.4 0.001	18.46 ± 4.89 19.18 ± 5.72 18.29 ± 6.03 13.6 ± 3.73 0.004	20.62 ± 5.82 19.81 ± 5.5 19.05 ± 6.21 18.25 ± 5.51 0.3
Gender	Female Male P-value*	17.92 ± 9.38 16.66 ± 8.65 0.34	26.84 ± 5.33 27.01 ± 4.64 0.69	18.72 ± 5.6 17.71 ± 5.58 0.2	19.97 ± 5.75 18.6 ± 6.12 0.23
Marital status	single Married P-value*	18.79 ± 10.96 17.31 ± 8.51 0.45	26.41 ± 6.06 27.02 ± 4.84 0.25	17.83 ± 5.51 18.75 ± 5.64 0.24	19.75 ± 6.36 19.64 ± 5.66 0.92
Having children	yes No P-value*	17.38 ± 9.18 17.8 ± 9.31 0.66	27.27 ± 4.92 26.20 ± 5.56 0.047	18.88 ± 5.87 17.75 ± 5.09 0.08	19.31 ± 6.06 20.13 ± 5.61 0.20
Having disease	yes No P-value*	17.58 ± 9.11 17.61 ± 9.30 0.98	26.9 ± 5.69 26.86 ± 5.12 0.77	17.06 ± 6.33 18.68 ± 5.48 0.06	18.44 ± 6.3 19.8 ± 5.82 0.14
Level of education	Undergraduate Bachelor MA and above P-value**	14.76 ± 8.72 18.06 ± 9.37 16.67 ± 8.85 0.26	26.86 ± 5.77 26.43 ± 5.29 28.69 ± 4.04 0.009	17 ± 5.1 18.83 ± 5.54 17.48 ± 6.1 0.12	17.63 ± 6.62 20.16 ± 5.68 18.17 ± 6.12 0.06
Work experience (years)	1–10 11–20 21–30 > 30 P-value**	18.8 ± 9.34 18.08 ± 9.56 12.72 ± 6.62 17.5 ± 9.27 0.001	23.06 ± 5.29 27.45 ± 5.16 27.41 ± 4.76 28.5 ± 0.07 0.2	18.93 ± 5.45 18.69 ± 5.98 16.39 ± 5.11 19 ± 4.24 0.07	20.21 ± 5.6 19.26 ± 6.12 18.95 ± 6.2 20.5 ± 7.77 0.42
Type of hospital	Public Private Semiprivate P-value**	17.30 ± 9.57 16.83 ± 9.27 19.25 ± 7.98 0.08	26.52 ± 5.4 29.08 ± 4.68 27.28 ± 4.38 0.04	18.16 ± 5.74 17.45 ± 4.99 19.76 ± 5.32 0.11	19.74 ± 6 19.16 ± 5.52 19.36 ± 5.66 0.72

* U-Mann-witney test

** Kruskal-Wallis test

The health anxiety of nurses aged between 31 and 40 years was significantly higher than nurses aged 51–60 years (P = 0.045). There was a significant weak inverse correlation between health anxiety score and age (r = -0.14; P = 0.002). Nurses aged 41–50 years used more problem-oriented strategies than younger nurses (P < 0.01). There was a significant positive correlation between problem-oriented strategies and age (r = 0.22; P < 0.001). The score of emotion-based strategies in nurses aged 51–60 years was significantly lower than in other age groups (P < 0.01).

The score of health anxiety in nurses who had a work experience of 21–30 years was significantly lower than nurses with less work experience (P < 0.001). There was a significant weak inverse correlation between health anxiety score and work experience (r = -0.18; P = 0.002).

The score of problem-oriented strategies was significantly higher in nurses who had children (P < 0.05), had master’s degree or higher (P = 0.001), and nurses working in private hospitals (P < 0.05) than in nurses who did not have children, had bachelor degree and worked in public hospitals.

There was no significant difference between the scores of avoidance-oriented strategies and any of the demographic variables (Table 4).

## Discussion

In this study, nurses’ health anxiety scores were high and the majority of nurses have health anxiety. Since our study was conducted on frontline nurses who directly cared for COVID patients, it is reasonable that the level of health anxiety in these nurses is high. Consistent with this finding, in other studies, the level of health anxiety of the medical staff, especially the frontline nurses, was high [9, 17, 18, 50]. Frontline nurses face much more psychological stress due to their work environment. Concerns about infection during close contact with patients, fear of transmitting the disease to the family, fear of skin damage caused by protective equipment, and observation of the suffering and death of the patient cause severe anxiety for nurses [3, 51, 52].

According to the results, the level of health anxiety of younger and less experienced nurses is higher than older and more experienced nurses. This finding is consistent with the findings of previous research [33, 53–56]. Young nurses may experience the epidemic for the first time and may be more anxious due to inexperience [57]. more experienced nurses have faced similar epidemics before, so they have more knowledge, skills, and self-regulation, are more adaptable, and are less anxious than less experienced nurses [58].

In this study, nurses mostly used problem-coping strategies to deal with anxiety caused by COVID-19. Consistent with this finding, in one study, the main strategies that frontline nurses demonstrated to cope with COVID-19 related stress were problem-oriented strategies including taking preventative measures, actively learning about COVID-19 and professional knowledge, setting a positive attitude about the COVID-19 epidemic, and chatting with family and friends [6].

In the present study, older nurses used more problem-oriented strategies. The relationship between age and coping strategies in other studies was reported with different results and there was no agreement between the studies. In some studies, older age was associated with problem-oriented strategies [59, 60] and in some studies, younger age was associated with problem-oriented strategies [61]. Older people seem to consistently experience less anger than their younger counterparts. The results of Yeung and Fung’s study showed that younger people used more emotion-focused coping than middle-aged and older people at the peak of SARS [59].

In this study, nurses with master’s degree or higher used problem-oriented strategies significantly more than bachelor nurses. This finding supports the results of previous studies [62, 63]. Higher education is usually associated with higher levels of mental function, which can help to better cope with stress [60]. Similar to the findings, in the study of Ferreira et al., significant positive correlations were reported between age and education with resilience [66]. Based on this, it seems that increasing age and education, by promoting resilience and controlling more stress, allow people to use more problem-oriented strategies compared to emotion-oriented and avoidance.

In the present study, nurses who had children and worked in private hospitals were more problem-oriented. Although other studies have reported different results on the relationship between having children, stress, and resilience in nurses, the results of Mealer et al.‘s study showed that parent nurses had more resilience and less stress than other nurses [64].

Nurses’ working conditions and stressors in public and private hospitals are different [65] and this factor can affect their coping strategies. Compatible with the findings of the present study, that nurses in private hospitals were more problem-oriented, in Fathi’s study, most nurses in public hospitals used emotion-based and ineffective coping strategies [66].

In this study, we investigated the correlation between COVID-19-related health anxiety and coping styles in frontline nurses. There was a positive significant correlation between the scores of health anxiety and emotion-oriented coping style. Nurses with higher health anxiety were more likely to use emotion-oriented strategies. This finding is consistent with the results of other studies in this field [3, 33, 47–50]. Mohammadzadeh et al. (2020) reported in their study that participants who used emotion-based coping strategies were four times more likely to have higher severity of anxiety than participants who used problem-based strategies. Folkman and Lazarus state that if people find stressors controllable, they are more likely to focus on the problem, otherwise, they are more focused on the emotion [51]. Therefore, when people find the situation uncontrollable and become highly anxious, they are more likely to use emotion-oriented strategies which are ineffective.

According to the results, the research hypothesis that there is a relationship between various coping strategies and health anxiety is accepted only for the emotion-oriented strategy. Since the scores of emotion-oriented strategies were higher in nurses who had health anxiety, therefore, emotion-oriented strategies cannot be an effective coping strategy to reduce health anxiety caused by COVID-19 in frontline nurses.

### Relevance for clinical practice

This study provides evidence that health anxiety is high in frontline nurses, especially in younger nurses with less work experience, and on the other hand, these nurses use more emotion-based coping strategies, which is one of the ineffective strategies. Therefore, nursing managers should consider training programs to control anxiety and improve coping skills in nurses, especially young and inexperienced nurses, and support them, in times of crisis and epidemic. According to the results of this study, front-line nurses, especially older age and more educated nurses, used more problem-oriented strategies. Therefore, the use of older and more educated nurses on the COVID-19 front line is recommended.

### Limitations and strengths

This study had some limitations. First, the inherent limitation of cross-sectional research makes it difficult to conclude a causal correlation. Second, sampling is done by convenience method that may produce selection bias. Third, we tried to collect data from different private and public hospitals, but the number of participants from each hospital was not equal, which may affect the generalizability of the results. Fourth, various factors such as personality traits can affect health anxiety and coping strategies. In this study, it was not possible to investigate all the influencing factors. Further studies are needed to examine the factors influencing the relationship between COVID-19-related health anxiety and coping strategies.

This study was the first to examine the correlation between health anxiety and coping strategies in Iranian nurses who directly cared for patients with Covid-19. In this study, standard and validated questionnaires were used to assess the level of health anxiety and coping strategies; The sample size was acceptable compared to other studies. In this study, bedsides investigating the relationship between health anxiety and coping strategies, demographic and occupational factors related to these variables were also examined and reported.

## Conclusion

According to the results of this study, COVID-19-related health anxiety was high in front-line nurses and the majority of nurses had health anxiety. Also, nurses with high health anxiety were more likely to use emotion-based coping strategies, which are ineffective. Consequently, it is necessary to pay more attention to the mental health of nurses, especially in epidemic conditions. Considering training programs to control health anxiety and improve coping skills in nurses, especially young and inexperienced nurses, and support them is recommended.

## Data Availability

The datasets used or analyzed during the present study are available from the corresponding author upon reasonable request. The data are not publicly available due to privacy and ethical restrictions.

## Abbreviations

COVID-19: Coronavirus disease 2019
